# Effect of Ciliates in Transfer of Plasmid-Mediated Quinolone-Resistance Genes in Bacteria

**DOI:** 10.3201/eid2103.141549

**Published:** 2015-03

**Authors:** Jose Luis Balcázar

**Affiliations:** Catalan Institute for Water Research, Girona, Spain

**Keywords:** gene transfer, antibiotic resistance, qnr genes, bacteria, quinolone resistance, plasmid-mediated resistance, PMQR genes, antimicrobial resistance

**To the Editor:** Previous studies have suggested that protozoa may promote horizontal gene transfer among bacterial species (*1*,*2*). This process is largely, although not exclusively, responsible for increasing the incidence of antibiotic-resistant bacteria through various mechanisms, such as transformation by acquisition of naked DNA, transduction by acquisition of DNA through bacteriophages, and conjugation by acquisition of DNA through plasmids or conjugative transposons (*3*,*4*). Because antibiotic resistance may be mediated by horizontal gene transfer, it is necessary to understand whether protozoa, which are widely distributed in nature, facilitate the acquisition and spread of antibiotic resistance genes. The aim of this study was to explore whether the ciliated protozoan *Tetrahymena thermophila* promotes the transfer of plasmid-mediated quinolone-resistance (PMQR) genes in bacteria.

Two *qnr* gene–positive bacterial strains (*Klebsiella oxytoca* and *Escherichia coli*) were chosen as donors, and azide-resistant *E. coli* strain J53 was used as a recipient for the assessment of gene transfer frequency. The *K. oxytoca* and *E. coli* strains were previously isolated and identified from the Ter River (Ripoll, Spain) in the framework of a multidisciplinary study on antibiotic-resistant bacteria (*5*). Donor and recipient bacteria, previously grown in Luria-Bertani broth for 5 h at 37°C, were mixed in equal numbers (10^9^ CFU/mL) with or without *T. thermophila* strain SB1969 (10^5^ cells/mL) in Page’s amoeba saline for 24 h, as previously described (*1*). Heat-treated ciliates, exposed for 10 min at 90°C, were also tested to determine whether viable organisms are required for gene transfer. Conjugation experiments were performed at 37°C, and, after the incubation period, the cultures were treated as previously described (*1*). Transconjugants were then selected on Luria-Bertani agar plates containing sodium azide (100 mg/L) and nalidixic acid (6 mg/L). The gene transfer frequency was estimated as the number of transconjugants for each recipient. Antibiotic susceptibility tests were also determined by using the broth microdilution method according to Clinical and Laboratory Standards Institute guidelines (*6*). All data were derived from >3 independent experiments, and statistical analyses were performed by using analysis of variance modeling, in which p<0.05 was considered significant (SPSS 17.0 software; IBM, Chicago, IL, USA).

The results revealed that the frequency of gene transfer between bacteria exposed to ciliates increased significantly (p<0.05), from 1.5 × 10^−7^ to 2.8 × 10^−6^ and from 1.2 × 10^−7^ to 1.6 × 10^−6^ in *E. coli* transconjugants of *K. oxytoca* (*qnrB*-positive strain) and *E. coli* (*qnrA*-positive strain), respectively (Figure). However, there were no differences in MIC values of ciprofloxacin and ofloxacin between transconjugants obtained from cultures exposed to ciliates and those from untreated cultures. These results suggest that, even though ciliates promote the transfer of PMQR genes, they did not induce increased expression of these genes. Moreover, no statistically significant differences were found in gene transfer efficiency between cultures exposed to heat-treated ciliates and those not exposed to ciliates. This finding suggests that the cell components of ciliates do not promote gene transfer and, therefore, other mechanisms may be responsible for this phenomenon. In fact, the presence of ciliates may increase the frequency of gene transfer by facilitating contact between donor and recipient bacteria through co-accumulation in their vesicles (*1*). Because protozoa are widely distributed in diverse environments, they may constitute a key environmental reservoir for acquisition and spread of antibiotic-resistance genes among bacteria, including human pathogens.

**Figure:**
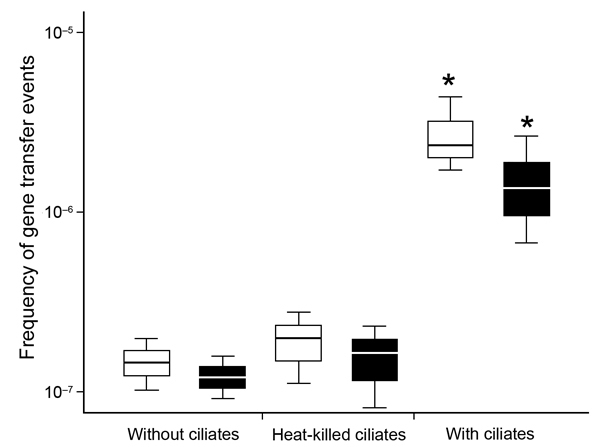
Box plot chart showing effects of ciliates on the transfer frequency of plasmid-mediated quinolone-resistance genes between *Escherichia coli* strain J53 and *qnrB*-positive *Klebsiella oxytoca* strain (white boxes) or *qnrA*-positive *E. coli* strain (black boxes). Box plots are divided by medians (black or white bars) into upper quartile and lower quartile ranges. Error bars indicate minimum and maximum values. Asterisks indicate a statistically significant difference (p<0.05) between treated and untreated cultures.

The study findings demonstrate that ciliates increase the transfer of PMQR genes in bacteria. These findings may therefore have important public health implications because the presence of ciliates would promote the spread of antibiotic resistance genes among bacterial species. According to recent data from the European Centre for Disease Prevention and Control (http://www.ecdc.europa.eu/), each year, ≈25,000 persons in the European Union die as a direct result of antibiotic-resistant infections. Thus, further studies are needed to determine the role of protozoa, such as ciliates, in the emergence and spread of antibiotic-resistant bacteria and to inform the implementation of appropriate public health strategies, policies, and mitigation programs. Elucidation of the mechanisms involved could lead to a better understanding of why some protozoa can promote gene transfer between bacteria.
